# The Impact of Internet Use on Health Outcomes of Rural Adults: Evidence from China

**DOI:** 10.3390/ijerph17186502

**Published:** 2020-09-07

**Authors:** Lili Li, Yiwu Zeng, Zhonggen Zhang, Changluan Fu

**Affiliations:** 1 China Academy for Rural Development, Zhejiang University, Hangzhou 310058, China; 11620059@zju.edu.cn (L.L.); zhgzhang@zju.edu.cn (Z.Z.); 2 School of Economics and Management, Hangzhou Normal University, Hangzhou 311121, China

**Keywords:** internet use, health outcomes, information accessibility, social interaction, rural China

## Abstract

Health, as basic human capital, is quite important for rural adults. However, in China, the average level of public health facilities and services is far lower in rural areas than in cities. In recent years, the internet has developed rapidly in China, and is increasingly affecting rural adults in a positive way. The purpose of this paper is to reveal whether internet use can be an effective way to improve the health of rural adults. This study used three rounds of data from the China Family Panel Studies (CFPS) collected in 2014, 2016, and 2018. After eliminating samples due to attrition, the study included 7528 villagers who were at least 16 years old. A panel logit model was employed to conduct an empirical analysis. The results indicate that internet use has a significantly positive impact on health outcomes of rural adults. By using the internet, rural adults can find a large amount of health information, increase their social interaction, and maintain physical exercise to improve their health. Thus, it is important to promote internet use for health purposes in rural areas. In addition, internet use had heterogeneous effects on the health of rural adults of different genders, age groups, and education levels. Attention should be focused on highly educated older men to improve the effects of internet use.

## 1. Introduction

As human capital theory suggests, health is an important component of human capital [1], and healthy human capital plays a huge role in promoting national economic growth [2]. In particular, people with a higher level of health can promote labor market participation and provide a stable labor force [3], which is an important condition for high income and economic status [4]. Human capital theory also points out that governments and individuals need to invest in health in order to have good health status [1]. For example, the government has the responsibility to provide public health services, such as sound infrastructure, regular hospitals, and comprehensive medical insurance systems [5], so that people have the ability to access high-quality services when they have health problems. Individuals need to master health knowledge and maintain high health literacy to manage their health.

Rural adults with good health status help to promote economic and social development in rural China, as well as the coordinated development of urban and rural areas, as rural adults constitute an important part of the labor force. However, chronic disease prevalence and other indicators show that the overall health status of rural Chinese adults has been declining. Specifically, the two-week prevalence rate for rural adults increased from 13.9% in 2003 to 20.2% in 2013. In addition, there is a large gap between urban and rural health expenditures, in terms of both public and private expenditure [6]. Since the founding of the People’s Republic of China, the government has been implementing preferential policies in favor of urban areas. Many resources are invested in urban areas, while rural areas lack support for development. Due to the unequal distribution of health service resources, rural adults have to travel long distances and pay transportation costs in order to access high-quality health services [7], which imposes a great burden. Moreover, results from the National Resident Health Literacy Survey show that in 2019, the health literacy level of urban adults was 24.81%, while that of rural adults was 15.67%, thus the average level of health literacy is lower in rural areas than in cities. In this context, how to improve the health status of rural adults, reduce inequalities in health, and achieve integrated development of urban and rural areas have become some of the issues of most concern for the Chinese government.

In the past two decades, the internet has developed rapidly. This rapid expansion has increased people’s access to health information, especially residents in rural and remote areas [8,9,10]. As of March 2020, China had 904 million internet users, and the internet penetration rate was 64.5% [11]; further, 33.2% of Chinese adults have sought health information from the internet [12]. In addition, the government has successively proposed a series of normalization strategies such as Internet+ and digital villages to develop rural areas. Specifically, in 2018, the State Council issued “Opinions on Promoting the Development of ‘Internet + Medical Health’” in order to promote deep integration, which helps in having access to health information, adopting a healthy lifestyle, and improving the accessibility of medical and health services for rural adults by using the internet. Therefore, the internet could help reduce social inequalities in health [13]. In this context, it is necessary and significant to explore whether and how the internet plays a role in improving the health of adults in rural China. Moreover, there are few studies directly focused on this topic. To fill that research gap, this study used three rounds of data from China Family Panel Studies (CFPS) collected in 2014, 2016, and 2018 to investigate the relationship between internet use and health outcomes of rural adults who are at least 16 years old. The study confirms that internet use has a significantly positive impact on rural adults’ health. Information accessibility, social interaction, and exercise are three important pathways linking internet use to improved health outcomes. In addition, internet use has heterogeneous effects on the health of rural adults of different genders, age groups, and education levels. Attention should be focused on highly educated older men in order to improve the effects of internet use. This study highlights the role of the internet in improving the health of rural adults, and thus contributes to both the field of internet use and rural human capital theory, and provides empirical evidence for accelerating the popularity of the internet in rural China.

The remainder of this paper is organized as follows. Section 2 reviews the existing literature. Section 3 explains data sources and the methodological approach of the study. The empirical results are presented and discussed in Section 4 and Section 5, respectively. Section 6 concludes the paper.

## 2. Literature Review

In recent years, a number of research efforts have empirically studied the impact of the internet on individual health, but results are mixed. On one hand, many studies found that internet use contributed to positive health outcomes of adults [9,14,15,16,17,18,19,20,21,22,23,24,25,26,27,28]. On the other hand, some found that internet use had no impact or a negative impact on health outcomes [17,19,29,30,31]. In general, most research suggests that the effect of the internet on adults’ health is positive, and those studies provide important references for the framework of this study.

The internet has the potential to improve adults’ health by providing public access to large amounts of health information, medical resources, and social support [9,14]. Compared with offline information resources, the internet has more advantages in terms of obtaining health-related information [20,21]. The internet enables the dissemination of health information, overcoming geographical and time constraints, making it more convenient and faster. The anonymity of the internet allows a greater variety of health information to be shared [22]. Internet use for health-related purposes could increase adults’ medical knowledge, improve their health literacy [15], and help them implement healthy behaviors, such as doing moderate exercise every week, eating healthy, and avoiding unhealthy behaviors [16]. It is found that internet users are more likely than non-internet users to have weekly moderate physical activity and eat healthy, and are less likely to smoke [17]. Moreover, the internet can provide opportunities for rural residents to interact with healthcare providers, especially for people in remote areas [9,18,23,24]. The use of the internet can also enhance communication with distant relatives and friends, leading to a larger and more useful social network [25,26], which is associated with better psychological functioning and well-being [19]. In addition, internet use for health helps people find social support from online support groups, which is conducive to their health-related knowledge and physical and mental health [19,27,28].

Although researchers have recently focused on issues of the relationship between internet use and health outcomes, some gaps exist. First, most research has studied the impact of seeking health information on the internet on adults with specific illnesses or diseases [32] or explored factors that predict internet use for health purposes [33]. Little is known about the impact of internet use by adults in general on health outcomes. Second, little research has empirically examined the pathways through which internet use could affect health outcomes. Third, little research has examined the issue with nationally representative data collected in recent years in rural China, where medical resources are scarce and adults have low health literacy. To bridge these gaps in the literature, this study uses longitudinal data from a nationwide survey to investigate the causal relationship between internet use and adults’ health outcomes in rural China. Specifically, this study examines whether and how internet use positively affects their health outcomes. On the basis of the existing literature mentioned above, we proposed three pathways—information accessibility, social interaction, and moderate exercise—through which internet use could improve health status. This study also examines whether these effects differ for people with different characteristics (for example, gender, age, and education). In addition, this study employs instrumental variables and the linear two-stage least squares (2SLS) method to address potential endogenous problems due to omitted variables or reverse causality.

## 3. Data and Method

### 3.1. Sample Selection

The study used data from the China Family Panel Studies (CFPS), which is a national and longitudinal survey conducted by the Institute of Social Science Survey (ISSS) at Peking University. The CFPS surveys economic and social development and changes in 25 provinces in China, excluding Tibet, Qinghai, Xinjiang, Ningxia, Inner Mongolia, Hainan, Hong Kong, Macau, and Taiwan. It uses a stratified, multi-stage sampling strategy to ensure that the sample represents 95% of the total population of China [34]. Therefore, the CFPS sample can be regarded as a nationally representative sample.

The first round of data collection was carried out in 2010, followed by a further four rounds in 2012, 2014, 2016, and 2018. Our study primarily used data from 2014, 2016, and 2018 since the CFPS not only contains an extensive set of measures of internet access and usage [34], but also uses the same set of test questionnaires to measure health outcomes and sociodemographic characteristics needed in our study. Moreover, this study is based on rural adults aged 16 years or older. The original sample of CFPS 2014 included 17,883 villagers and 7042 rural households in 192 counties (districts) in 25 provinces. CFPS followed up with 8091 villagers in 2016 and 2018. After eliminating samples with missing values and inconsistent personal information, the final sample included 7528 villagers.

### 3.2. Data Collection

#### 3.2.1. Outcome Measurement

The dependent variable in this study is the self-reported health outcomes of rural adults, as measured by a question about asking the health condition of respondents in the CFPS. The CFPS measures it on a scale from 1 to 5, with 1 indicating extremely healthy, 2 indicating very healthy, 3 indicating relatively healthy, 4 indicating general healthy, and 5 indicating unhealthy. Self-reported health is a comprehensive assessment of a respondent’s own health based on the severity of disease, family disease history, and stability of health status. This measurement method satisfies the adequacy of psychometrics and the reliability and validity of statistics. Thus, many current studies on adult health use similar measurement methods [35]. In addition, the respondents can clearly distinguish whether they are healthy or unhealthy, but they cannot know exactly what level of health they have reached. Therefore, some scholars propose using dummy variables to measure self-reported health outcomes [35,36]. This study learned from this approach. Specifically, this study transformed these answers into dummy variables, defining health outcomes with values of 1 to 4 as healthy and assigning these a new value of 1, and defining a value of 5 as unhealthy and assigning it a new value of 0.

#### 3.2.2. Internet Use Measurement

The main set of independent variables in the analysis is internet use, which is measured in two ways: general usage and weekly online time. General internet usage is a binary variable (1 = yes, 0 = no) that is determined by asking whether respondents had access to the internet (in any capacity and via any means) in the CFPS survey. Weekly online time (hours) is a continuous variable measured by asking respondents how many hours they spend online each week in their spare time.

#### 3.2.3. Control Variables

The following covariates were used to control potential confounding in the relationship between internet use and health outcomes of rural adults. First, this study controlled individual characteristics: gender, age, education, marriage, exercise (does the person exercise every week), smoking (did the person smoke in the past month), drinking (did the person drink alcohol more than three times a week in the past month), sleep quality (how many hours of sleep each night), and work (does the person have a job). Second, several household characteristics were controlled for: number of family members, household income per capita, number of houses owned, and family gift exchange (the sum of income and expenses for gifts and cash for family social activities). In the regression analysis, we took the logarithm of household income per capita and family gift exchange.

The baseline characteristics of rural adults in our sample are shown in Table 1. In all, 48.2% of respondents were men and 51.8% were women, 95.7% were married, and 80.0% had paid work. The average age of respondents was 49 years. With respect to education, the average years of education was 5.8, indicating that the education level of rural adults was low and below primary school. It can be seen that 25.6% of respondents exercised weekly, 31.7% smoked, and 16.4% had drunk alcohol more than three times a week in the past month. The average sleep duration every night was 7.9 h. The majority of respondents (80.3%) were in good health. However, the percentage of rural adults using the internet was 13.7% in 2014, which was far lower than the national overall internet penetration rate of 47.9% [37]. Further information about the household characteristics of respondents are provided in Table 1.

### 3.3. Analytical Method

This subsection describes our analytical approach. Since health is a binary dummy variable, this study constructed a panel logit model with robust standard error clustering at the village level to study the impact of internet use on health outcomes. The specific model is as follows:

(1) $$P(y_{it}=1|x_{it},\beta ,\mu_{i})=\Phi (\mu_{i}+x_{it}\beta )=\frac{e^{\mu_{i}+x_{it}\beta }}{1+e^{\mu_{i}+x_{it}\beta }}.$$

In this equation, $y_{it}$ is the outcome variable representing the health status of villager i at time t, $x_{it}$ is a vector of variables that capture internet use measured by general usage and weekly online time and other control variables including individual characteristics and household characteristics, $\mu_{i}$ is the individual effect, and Φ(⋅) denotes cumulative distribution function subject to logistic distribution.

## 4. Results

Whether and how internet use significantly and positively affect health outcomes of rural adults was empirically tested. To do this, multivariate analysis was first adopted to examine the impacts of internet use (measured by general usage and weekly online time) while controlling for individual and household characteristics. Second, a potential path analysis for the impacts was conducted. Third, whether internet use has different impacts on health outcomes of rural adults with different characteristics was examined. Finally, the causal relationship between internet use and health outcomes was tested by solving the endogenous problem.

### 4.1. Basic Regression

The results for the impact of internet use on health outcomes of rural adults are found in Table 2 and are reported in standard deviations (SD). Table 2 shows a positive and significant effect of general internet usage on health outcomes (columns 1 and 2). Specifically, in the regression without the year dummy variable, health status improved by 0.266 standard deviations at the 0.01 level of significance (row 1, column 1) if a rural resident used the internet. In the regression including the year dummy variable, health status improved by 0.351 standard deviations (row 1, column 2, *p* < 0.01), which is greater than the effect without the year dummy variable. Similar results can be found from another measure of internet use, weekly online time (columns 3 and 4). It can be seen that if rural adults increased their weekly online time by one hour, their health outcomes would improve by 0.010 standard deviations (row 2, column 3) in the regression without the year dummy variable. The coefficient was 0.012 (row 2, column 4) after including the year dummy variable. All four regressions show that internet use has a positive and significant impact on health outcomes of rural adults. After controlling for the year dummy variable, internet use had a greater effect on health outcomes. This is consistent with previous research showing that health outcomes of rural adults are improved by using the internet [9].

Table 2 also shows that men who were married, younger, and more educated were more likely to report good health. Individuals who exercised weekly and did not smoke and drink were significantly more likely to report good health. Only after controlling for the year dummy variable was sleep quality significantly correlated with health outcomes. The coefficients in columns 2 (0.033) and 4 (0.032) are both positive and significant at the 0.1 level, but small in size. In addition, people who had a job, more family members, more houses, and higher household income reported better health conditions. All of these effects are statistically different from zero.

### 4.2. Pathway Examination

This study examined three potential pathways linking internet use to improved health outcomes. One path is that internet use allows people to have access to a large amount of health information conveniently, which is positively associated with health status [38]. Using the internet to seek health information helps in receiving necessary informational support that improves people’s ability to deal with health-related problems [39]. Internet use also has direct positive paths to social interaction and social support, which, in turn, are positively associated with health outcomes [40]. Social interaction online can help rural adults get support and advice, improve their health knowledge, and manage their health, leading to better health outcomes [41]. In particular, internet use can expand people’s social networks and increase their social interactions to obtain social support resources from distant friends, online support communities, and healthcare experts [19]. Another path is that internet use can increase people’s health knowledge and promote health behavior changes [16], such as engaging in weekly physical activity and not smoking. People who were nonsmokers and did exercise weekly were significantly more likely to report good health [38].

In addition, there are relevant questions to measure information accessibility, social interaction, and exercise in the CFPS survey. Information accessibility is a categorical variable captured by a question in the CFPS that asks how important using the internet is to obtain information. Respondents gave their answers on a 5-point Likert scale (from 1 = very unimportant to 5 = very important). Social interaction is a continuous variable measured by family gift exchange, which is the sum of all income and expenses of gifts and cash for family social activities. Information about incomes and expenses of family gifts and cash is collected in the CFPS survey. Exercise is measured by asking respondents how many times a week they do exercise. In this paper, the answers are transformed into a dummy variable, defining weekly exercise with a value of 1 and no exercise with a value of 0. In the regression of information accessibility on internet use, this study used an ordered logistic model, since information accessibility is an ordered discrete variable. In the regression of social support, the random effects model was employed.

As shown in Table 3, the analysis examined the impacts of internet use on three paths: information accessibility, social interaction, and exercise. It can be seen that general internet usage is positively associated with information accessibility (row 1, column 1, *p* < 0.01), which means that compared with people who do not use the internet, users claim that the internet is an important channel for obtaining information. The same results were found in the regression of weekly online time (row 2, column 2). When rural adults are online for more time each week, they are more likely to obtain health information on the internet, thus helping to improve their health. All of these coefficients are significantly different from zero. Columns 3 and 4 show that internet use (general usage and weekly online time) also has a positive impact on social interaction. However, it can be seen that the effect of weekly online time (row 2, column 4) is not statistically different from zero. In other words, as predicted by a number of authors in the literature [19], the more time rural adults spend online to increase their social networks, the less time they have to maintain and strengthen their social relationships offline, which may not necessarily improve overall social relationships. Finally, this study examined the impact of internet use on rural adults’ exercise. Similar to the regression of information accessibility, general internet usage (coefficient = 0.546; row 1, column 5) and weekly online time (coefficient = 0.017; row 2, column 6) both help to encourage rural adults to exercise moderately every week. Moreover, these effects are significant at the 0.01 level and moderate in size.

### 4.3. Heterogeneous Effects

To further draw out the important effects of internet use on health outcomes, this study examined important subgroups of rural adults, specifically, gender, age, and education groups. The effects of internet use on rural adults of different genders are presented in Table 4. It can be seen that general internet usage (coefficient = 0.538; row 1, column 3) and weekly online time (coefficient = 0.027; row 2, column 4) both had a greater impact on health outcomes for women. These coefficients are positive and significant at the 0.01 level. For men, the coefficients of general internet usage and weekly online time are small and not statistically different from zero, although general usage shows a positive impact on health outcomes (row 1, column 1). Moreover, weekly online time was negatively associated with men’s health status (row 2, column 2). This is consistent with the previous literature showing that women were more likely to use the internet to seek heath information [42], which had a positive impact on health status.

Considering the different impact of internet use on the health outcomes of rural adults of different age groups, as shown in Table 5, it can be seen that general internet usage had a positive and significant effect on health outcomes for rural adults in the 16–39 age group (row 1, column 1) and the 40–59 age group (row 1, column 3). The magnitude of this effect was larger for the former (0.461) than for the latter (0.212). However, the effect of general internet usage on people over 60 years old was the smallest and is not significant (coefficient = 0.081; row 1, column 5). This may be because young people are more likely to use the internet to seek heath information, and more experience with internet usage could enhance users’ attitude toward the technology and increase their capacity to take advantage of resources they find online [9,43], which in turn positively affects health outcomes [9]. In addition, the effects of weekly online time were not significant for rural adults in all age groups (row 2).

In Table 6, which presents the impact of internet use on health outcomes of rural adults with different education levels, it can be seen that the effects of internet use are significant only among people with primary school education or below. Specifically, general internet usage was positively associated with health outcomes of rural adults with primary school education or below (row 1, column 1), although the coefficient (0.462) is smaller than the coefficient (0.521) for people with education beyond senior high school. A similar result of the effect of weekly online time (coefficient = 0.023; row 2, column 2) can be seen, which is positive and significant. Again, the magnitude of this effect is smaller compared to people with education beyond senior high school (coefficient = 0.028; row 2, column 6). For people with an education level between junior and senior high school, the effect of internet use was not significant, and the size is the smallest (columns 3 and 4).

### 4.4. Endogenous Issues

Although this study used longitudinal data and a panel logit model, which allow stronger causal claims about the relationship between internet use and health outcomes than cross-sectional data [19], there may still be endogenous problems, and thus, an inability to draw a causal relationship between the two. The longitudinal design of the CFPS follows the same individuals over many years, which helps in controlling stable characteristics such as demographic differences and personality; however, it cannot control for unmeasured variables that may change over time. There may also be reverse causality between internet use and health outcomes, since people with poor health are more likely to use the internet to seek health-related information or find support resources. Therefore, our study used two instrumental variables (provincial internet penetration and whether there is a computer at home) to address endogenous problems. Internet penetration refers to the popularity and utilization of internet facilities in a region, which is not directly related to rural adults’ health. However, internet penetration has an important impact on individuals’ online decision-making, which satisfies the exogenous requirements of instrumental variables. Having a computer at home (1 = yes, 0 = no) also meets the requirements of instrumental variables. Specifically, whether people have a computer at home is positively associated with their internet use. Individuals who have a computer at home are more inclined to use the internet, although those who do not may also use smartphones or other people’s computers to surf the internet. On the other hand, whether people have a computer at home generally does not directly affect their health through channels other than the internet. A general fact is that if the computer at home is not connected to the internet, it is easy for it to be idle, since its functions are extremely limited, and it has little effect on the owner. Moreover, this study used the two-stage least squares (2SLS) method to estimate the effect of internet use on health outcomes.

Table 7 shows the results of the impact of general internet usage and weekly online time on health outcomes of rural adults using instrumental variables. In the first-stage regression, both instrumental variables had a significant impact on general internet usage (rows 1 and 2, column 1), which is positively and significantly associated with health outcomes (row 3, column 2). The coefficient (0.099) decreased by 0.252 after addressing the endogenous problem. Similar results can also be seen for the impact of weekly online time. Specifically, both instrumental variables are significantly associated with weekly online time (rows 1 and row 2, column 3) at a significance level of *p* = 0.01. Moreover, weekly online time had a significant effect on health outcomes (row 4, column 4). The effect (0.009) decreased by 0.003 after solving endogeneity.

## 5. Discussion

The findings of this study are consistent with most of the previous relevant research, showing that internet use has a positive and significant impact on health outcomes [9]. After addressing endogenous problems, internet use still has a positive and significant impact. Moreover, this study found that internet use is positively associated with information accessibility, which means that the internet is an important source of information for rural adults to access health information. This study also found that internet use increases social interaction significantly, by which people can obtain social support and helpful advice. In addition, internet use has a significant effect on people’s health behaviors. In particular, rural adults will do exercise every week to improve their health status. The findings showed that internet use provides more opportunities for rural adults to seek health information, draw on more social support resources, and have better health behaviors, which help to improve their health literacy and enhance their ability to manage their health, leading to better health outcomes.

Another important finding is that internet use has heterogeneous effects on the health of rural adults of different genders, age groups, and education levels. In the regression that shows gender heterogeneity, the results demonstrate that internet use has a positive and significant impact on women’s health outcomes, with greater effects than on men’s health outcomes. However, the effect of internet use on men’s health was not significant. For rural adults of different age groups, it was found that the health of rural adults in younger age groups could be improved by general internet use. Specifically, there are significant and positive effects of general internet usage on health outcomes of rural adults in the 16–39 and 40–59 age groups, and the effect size for the former is almost twice that of the latter. However, this study did not find a significant relationship between general internet use and health outcomes of people aged 60 or above. This may be because older people are less likely to use the internet, and they have low internet skills, which means they are unable to benefit from online health resources [33], especially in rural China. In addition, this study found that internet use only had a positive and significant effect on the health outcomes of people with primary school education or below. This may be because rural adults with more education generally have high socioeconomic status, and they have more opportunities to communicate directly with healthcare providers and obtain health resources.

Despite these findings, in the future, attention should be paid to digital skills, which play a key role in using the internet effectively to seek health resources, increasing people’s medical knowledge, and managing their health. However, most people in rural China are older and have lower socioeconomic status and digital skills, and thus are unable to take full advantage of online health resources [13,33]. Moreover, rural adults have great difficulty in identifying correct online health information, as they generally have low health literacy, and thus, they cannot search for and understand the applicable information [44]. There are disadvantaged people who do not possess the necessary skills to search for health information online, which may create inequalities in health information accessibility for rural adults [33]. Attention should be paid to bridging the digital gap and maximizing the impact of the internet on people’s health. More research is warranted to further explore the relationship between digital skills and health outcomes.

In addition, our study did not explore the effect of different types of internet use on health outcomes, as each pattern is likely to have a different impact on people’s well-being, especially their physical and mental health. For instance, health-related internet use was positively associated with health status [9]. People can search for health information online and get social support from online communities and others who share similar experiences [30,45]. Moreover, the internet may affect health and well-being by affecting the ease with which people can access social support from friends and family [7,8], since most people are more likely to use the internet to communicate with distant friends and family members, which can strengthen already-existing social networks [46]. In addition, using the internet for entertainment and relaxation is to some extent regarded as a leisure activity, which helps people reduce stress and improve health outcomes [47]. Last but not least, the health outcomes were measured by people’s own reports of their general health. More objective measurements of health outcomes should be considered in future studies.

Despite these limitations, this study has important implications, which are particularly important in the context of rural China, which lags far behind urban areas. Information technology has developed rapidly and brought significant changes to personal life in recent years, especially health-related internet use. Our study found that internet use has a positive and significant impact on the health outcomes of rural adults. Rural adults can find a large amount of health information, increase their social interaction, and maintain physical exercise every week to improve their health outcomes by using the internet. Thus, it is important to promote internet use for health purposes in rural areas. Given that online health information is sometimes unreliable and rural adults have low health literacy, future research should focus on ways that not only encourage people to obtain health resources online, but also provide knowledge or resources to increase their ability to identify correct information efficiently. Health-promoting actions should be designed in terms of internet use. 

## 6. Conclusions

Although previous studies investigated the impact of internet use on people’s health outcomes, the causal relationship between the two is not yet fully understood. Most of the studies on this topic only used cross-sectional data or simple correlation analysis to investigate this relationship. Moreover, little research has examined the issue in developing countries, especially in rural China, where adults lack high-quality medical resources. To bridge the gaps in the literature, this study examined the causal relationship between internet use and health outcomes of rural adults using instrumental variables and longitudinal data from three waves of a nationally representative survey in rural China. The results indicate that internet use has a significantly positive impact on health outcomes of rural adults. This study also examined three pathways, information accessibility, social interaction, and exercise, through which internet use could improve health outcomes. In addition, this study investigated the heterogeneous effects of internet use on rural adults of different genders, ages, and education levels. Attention should be focused on highly educated older men to improve the effects of internet use.

## Figures and Tables

**Table 1:** Baseline characteristics of rural adults. SD: standard deviation.

Variable	Definition	Mean	SD
Dependent variable			
Health	1 = healthy, 0 = unhealthy	0.803	0.398
Independent variables			
General internet usage	1 = yes, 0 = no	0.137	0.344
Weekly online time	hours	1.339	5.445
Individual characteristics			
Gender	1 = male, 0 = female	0.482	0.500
Age	years	49.202	13.467
Education	years	5.799	4.249
Marriage ^a^	1 = yes, 0 = no	0.957	0.202
Exercise	1 = yes, 0 = no	0.256	0.437
Smoke	1 = yes, 0 = no	0.317	0.465
Drink	1 = yes, 0 = no	0.164	0.370
Sleep quality	hours per night	7.932	1.629
Work	1 = yes, 0 = no	0.800	0.400
Household characteristics			
Number of family members		4.446	1.917
Household income per capita	yuan	10,597.400	20,725.550
Number of houses owned		1.160	0.445
Family gift exchange	yuan	5282.456	10,228.840

^a^ Those who have never been married or are cohabiting are regarded as unmarried, while others are considered married.

**Table 2:** Impact of internet use on health outcomes of rural adults.

Variables	Dependent Variable: Health Outcome			
	(1)	(2)	(3)	(4)
General internet usage	0.266 ***	0.351 ***		
	(0.088)	(0.089)		
Weekly online time			0.010 *	0.012 **
			(0.006)	(0.006)
Gender	0.624 ***	0.555 ***	0.630 ***	0.563 ***
	(0.104)	(0.106)	(0.105)	(0.106)
Age	−0.070 ***	−0.065 ***	−0.072 ***	−0.067 ***
	(0.004)	(0.004)	(0.004)	(0.004)
Education	0.068 ***	0.090 ***	0.070 ***	0.093 ***
	(0.009)	(0.010)	(0.009)	(0.010)
Marriage	0.442 *	0.419 *	0.445 *	0.424 *
	(0.233)	(0.235)	(0.235)	(0.238)
Exercise	0.145 **	0.173 ***	0.156 **	0.181 ***
	(0.061)	(0.062)	(0.061)	(0.062)
Smoking	0.158 *	0.174 *	0.159 *	0.177 *
	(0.091)	(0.092)	(0.091)	(0.092)
Drinking	0.662 ***	0.660 ***	0.660 ***	0.657 ***
	(0.105)	(0.105)	(0.105)	(0.106)
Sleep quality	0.031	0.033 *	0.030	0.032 *
	(0.019)	(0.019)	(0.019)	(0.019)
Work	0.602 ***	0.610 ***	0.602 ***	0.610 ***
	(0.077)	(0.076)	(0.077)	(0.077)
Number of family members	0.088 ***	0.084 ***	0.087 ***	0.083 ***
	(0.019)	(0.019)	(0.019)	(0.019)
Household income per capita	0.037 ***	0.041 ***	0.038 ***	0.041 ***
	(0.013)	(0.013)	(0.013)	(0.013)
Number of houses owned	0.218 ***	0.215 ***	0.219 ***	0.216 ***
	(0.072)	(0.072)	(0.072)	(0.072)
Family gift exchange	−0.006	−0.008	−0.006	−0.008
	(0.005)	(0.005)	(0.005)	(0.005)
Year dummy variable	No	Yes	No	Yes
Constant	3.197 ***	2.618 ***	3.299 ***	2.799 ***
	(0.382)	(0.383)	(0.381)	(0.383)
Number of observations	22,584	22,584	22,584	22,584

***, **, * represent statistically significant at 1%, 5%, 10%, respectively.

**Table 3:** Regression results of pathway examination.

Variables	Information Accessibility		Social Interaction		Exercise	
	(1)	(2)	(3)	(4)	(5)	(6)
General internet usage	2.359 ***		0.385 ***		0.546 ***	
	(0.075)		(0.132)		(0.068)	
Weekly online time		0.103 ***		0.000		0.017 ***
		(0.005)		(0.007)		(0.003)
Year dummy variable	Yes	Yes	Yes	Yes	Yes	Yes
Control variables	Yes	Yes	Yes	Yes	Yes	Yes
Number of observations	22,584	22,584	22,584	22,584	22,584	22,584

*** represent statistically significant at 1%.

**Table 4:** Impact of internet use on health outcomes of women and men.

Variables	Men		Women	
	(1)	(2)	(3)	(4)
General internet usage	0.133		0.538 ***	
	(0.119)		(0.134)	
Weekly online time		−0.003		0.027 ***
		(0.008)		(0.009)
Year dummies	Yes	Yes	Yes	Yes
Control variables	Yes	Yes	Yes	Yes
Number of observations	10,896	10,896	11,688	11,688

*** represent statistically significant at 1%.

**Table 5:** Impact of internet use on health outcomes in different age groups.

Variables	16–39 Age Group		40–59 Age Group		>60 Age Group	
	(1)	(2)	(3)	(4)	(5)	(6)
General internet usage	0.461 *		0.212 *		0.081	
	(0.242)		(0.110)		(0.287)	
Weekly online time		−0.005		0.010		−0.000
		(0.011)		(0.008)		(0.023)
Year dummy variable	Yes	Yes	Yes	Yes	Yes	Yes
Control variables	Yes	Yes	Yes	Yes	Yes	Yes
Number of observations	4542	4542	11,182	11,182	6860	6860

* represent statistically significant at 10%.

**Table 6:** Impact of internet use on health outcomes of rural adults with different educational backgrounds.

Variables	Primary School and below		Junior and Senior High School		Senior High School and above	
	(1)	(2)	(3)	(4)	(5)	(6)
General internet usage	0.462 ***		0.273		0.521	
	(0.123)		(0.180)		(0.353)	
Weekly online time		0.023 **		−0.004		0.028
		(0.009)		(0.010)		(0.021)
Year dummy variable	Yes	Yes	Yes	Yes	Yes	Yes
Control variables	Yes	Yes	Yes	Yes	Yes	Yes
Number of observations	15,616	15,616	5235	5235	1733	1733

***, ** represent statistically significant at 1%, 5%, respectively.

**Table 7:** Results of instrumental variable (IV) regression.

Variables	First Stage	IV Regression	First Stage	IV Regression
	(1)	(2)	(3)	(4)
Internet penetration	0.109 **		2.549 ***	
	(0.049)		(0.760)	
Having a computer at home (1 = yes, 0 = no)	0.167 ***		2.015 ***	
	(0.009)		(0.193)	
General internet usage		0.099 **		
		(0.039)		
Weekly online time				0.009 ***
				(0.003)
Year dummy variable	Yes	Yes	Yes	Yes
Control variables	Yes	Yes	Yes	Yes
Number of observations	22,584	22,584	22,584	22,584

***, ** represent statistically significant at 1%, 5%, respectively.

## References

[1] Mushkin S.J. (1962). Health as an Investment. J. Political Econ..

[2] Fogel R.W. (1994). Economic Growth, Population Theory, and Physiology: The Bearing of Long-term Processes on the Making of Economic Policy. Am. Econ. Rev..

[3] Cai L. (2010). The Relationship between Health and Labor Force Participation: Evidence from a Panel Data Simultaneous Equation model. Labor Econ..

[4] Currie J. (2009). Healthy, Wealthy, and Wise: Socioeconomic Status, Poor Health in Childhood, and Human Capital Development. J. Econ. Lit..

[5] Balarajan Y., Selvaraj S., Subramanian S.V. (2011). Health Care and Equity in India. Lancet.

[6] Lv N., Zou W. (2015). Health Investment and Income: An Endogenous Growth Model and Empirical Analysis Based on Private and Public Health Investment. J. China Univ. Geosci. (Soc. Sci. Ed.).

[7] Ruishan H., Dong S., Zhao Y., Hu H., Li Z. (2013). Assessing Potential Spatial Accessibility of Health Services in Rural China: A Case Study of Donghai County. Int. J. Equity Health.

[8] Samkange-Zeeb F., Ernst S.A., Klein-Ellinghaus F., Brand T., Reeske-Behrens A., Plumbaum T., Zeeb H. (2015). Assessing the Acceptability and Usability of an Internet-Based Intelligent Health Assistant Developed for Use among Turkish Migrants: Results of a Study Conducted in Bremen, Germany. Int. J. Environ. Res. Public Health.

[9] Jiang S., Street R.L. (2017). Pathway Linking Internet Health Information Seeking to Better Health: A Moderated Mediation Study. Health Commun..

[10] Benvenuto M., Avram A., Sambati F.V., Avram M., Viola C. (2020). The Impact of Internet Usage and Knowledge-Intensive Activities on Households’ Healthcare Expenditures. Int. J. Environ. Res. Public Health.

[11] China Internet Network Information Center (CNNIC) (2020). The 45th China Statistical Report on Internet Development. http://www.cnnic.net.cn/hlwfzyj/hlwxzbg/hlwtjbg/202004/P020200428596599037028.pdf.

[12] Wang M.P., Viswanath K., Lam T.H., Wang X., Chan S.S. (2013). Social Determinants of Health Information Seeking among Chinese Adults in Hong Kong. PLoS ONE.

[13] Rains S.A. (2008). Health at High Speed: Broadband Internet Access, Health Communication, and the Digital Divide. Commun. Res..

[14] Murero M., Rice R.E., Murero M., Rice R.E. (2006). E-Health Research. The Internet and Health Care: Theory, Research, and Practice.

[15] Takahashi Y., Ohura T., Ishizaki T., Okamoto S., Miki K., Naito M., Akamatsu R., Sugimori H., Yoshiike N., Miyaki K. (2011). Internet Use for Health-Related Information via Personal Computers and Cell Phones in Japan: A Cross-Sectional Population-Based Survey. J. Med. Int. Res..

[16] Webb T., Joseph J., Yardley L., Michie S. (2010). Using the Internet to Promote Health Behavior Change: A Systematic Review and Meta-Analysis of the Impact of Theoretical Basis, Use of Behavior ChangeTechniques, and Mode of Delivery on Efficacy. J. Med. Int. Res..

[17] Xavier A.J., d’Orsi E., Wardle J., Demakakos P., Smith S.G., von Wagner C. (2013). Internet Use and Cancer-Preventive Behaviors in Older Adults: Findings from a Longitudinal Cohort study. Cancer Epidemiol. Biomark. Prev..

[18] Hao H. (2015). The Development of Online Doctor Reviews in China: An Analysis of the Largest Online Doctor Review Website in China. J. Med. Int. Res..

[19] Bessière K., Pressman S., Kiesler S., Kraut R. (2010). Effects of Internet Use on Health and Depression: A Longitudinal Study. J. Med. Int. Res..

[20] Trotter M.I., Morgan D.W. (2008). Patients’ Use of the Internet for Health Related Matters: A Study of Internet Usage in 2000 and 2006. Health Inform. J..

[21] Kontos E.Z., Emmons K.M., Puleo E., Viswanath K. (2012). Contribution of Communication Inequalities to Disparities in Human Papillomavirus Vaccine Awareness and Knowledge. Am. J. Public Health.

[22] Cotten S.R., Gupta S.S. (2004). Characteristics of Online and Offline Health Information Seekers and Factors that Discriminate between Them. Soc. Sci. Med..

[23] Oh H.J., Lee B. (2012). The Effect of Computer-Mediated Social Support in Online Communities on Patient Empowerment and Doctor-Patient Communication. Health Commun..

[24] Southwell B.G. (2013). Social Networks and Popular Understanding of Science and Health: Sharing Disparities.

[25] Wellman B., Haase A.Q., Witte J., Hampton K. (2001). Does the Internet Increase, Decrease, or Supplement Social Capital? Social Networks, Participation, and Community Commitment. Am. Behav. Sci..

[26] Katz J.E., Rice R.E. (2002). Social Consequences of Internet Use: Access, Involvement, and Interaction.

[27] Braithwaite D.O., Waldron V.R., Finn J. (1999). Communication of Social Support in Computer-Mediated Groups for People with Disabilities. Health Commun..

[28] Kummervold P.E., Gammon D., Bergvik S., Johnsen J.-A.K., Hasvold T., Rosenvinge J.H. (2002). Social Support in a Wired World: Use of Online Mental Health Forums in Norway. Nord. J. Psychiatry.

[29] Balsa A., Gandelman N. (2010). The Impact of ICT on Health Promotion: A Randomized Experiment with Diabetic Patients.

[30] Cline R.J.W., Haynes K.M. (2001). Consumer Health Information Seeking on the Internet: The State of the Art. Health Educ. Res..

[31] Eysenbach G., Powell J., Kuss O., Sa E.-R. (2002). Empirical Studies Assessing the Quality of Health Information for Consumers on the World Wide Web: A Systematic Review. JAMA.

[32] Gustafson D.H., Hawkins R., McTavish F., Pingree S., Chen W.C., Volrathongchai K., Stengle W., Stewart J.A., Serlin R.C. (2008). Internet-Based Interactive Support for Cancer Patients: Are Integrated Systems Better?. J. Commun..

[33] Jacobs W., Amuta A.O., Jeon K.C. (2017). Health Information Seeking in the Digital Age: An Analysis of Health Information Seeking Behavior among US Adults. Cogent Soc. Sci..

[34] Xie Y., Hu J. (2014). An Introduction to the China Family Panel Studies (CFPS). Chin. Sociol. Rev..

[35] Zhou G., Fan G., Shen G. (2014). The Income Disparity, the Social Capital and Health: A Case Study Based on China Family Panel Studies. Manag. World.

[36] Han L., Bai Y., Zhang L. (2019). Social Capital and Individual Health: Empirical Analysis Based on CFPS Data. J. Xiangtan Univ. (Philos. Soc. Sci.).

[37] China Internet Network Information Center (CNNIC) (2015). The 35th China Statistical Report on Internet Development. http://www.cnnic.net.cn/hlwfzyj/hlwxzbg/201502/P020150203551802054676.pdf.

[38] Suka M., Odajima T., Okamoto M., Sumitani M., Igarashi A., Ishikawa H., Kusama M., Yamamoto M., Nakayama T., Sugimori H. (2015). Relationship between Health Literacy, Health Information Access, Health Behavior, and Health Status in Japanese People. Patient Educ. Couns..

[39] Barak A., Boniel-Nissim M., Suler J. (2008). Fostering Empowerment in Online Support Groups. Comput. Hum. Behav..

[40] Beaudoin C.E., Tao C.C. (2008). Modeling the Impact of Online Cancer Resources on Supporters of Cancer Patients. New Media Soc..

[41] Ahmadi A. (2016). Social Support and Women’s health. Womens Health Bull..

[42] Ishikawa Y., Nishiuchi H., Hayashi H., Viswanath K. (2012). Socioeconomic Status and Health Communication Inequality in Japan: A Nationwide Cross-Sectional Survey. PLoS ONE.

[43] Chang H.H., Chen S.W. (2008). The Impact of Customer Interface Quality, Satisfaction and Switching Costs on E-loyalty: Internet Experience as a Moderator. Comput. Hum. Behav..

[44] Hernandez L.M. (2013). Health Literacy: Improving Health, Health Systems and Health Policy around the World: Workshop Summary.

[45] Preece J. (2000). Online Communities: Designing Usability and Supporting Sociability.

[46] Bessière K., Kiesler S., Kraut R., Boneva B.S. (2008). Effects of Internet Use and social Resources on Changes in Depression. Inf. Commun. Soc..

[47] Pressman S.D., Matthews K.A., Cohen S., Martire L.M., Michael S., Baum A., Schulz R. (2009). Association of Enjoyable Leisure Activities with Psychological and Physical Well-Being. Psychosom. Med..

